# Age and metabolic risk factors associated with oxidatively damaged DNA in human peripheral blood mononuclear cells

**DOI:** 10.18632/oncotarget.3202

**Published:** 2014-12-18

**Authors:** Mille Løhr, Annie Jensen, Louise Eriksen, Morten Grønbæk, Steffen Loft, Peter Møller

**Affiliations:** ^1^ Department of Public Health, Section of Environmental Health, University of Copenhagen, Copenhagen, Denmark; ^2^ National Institute of Public Health, University of Southern Denmark, Odense, Denmark

**Keywords:** 8-oxodG, comet assay, metabolic syndrome, obesity, oxidative stress

## Abstract

Aging is associated with oxidative stress-generated damage to DNA and this could be related to metabolic disturbances. This study investigated the association between levels of oxidatively damaged DNA in peripheral blood mononuclear cells (PBMCs) and metabolic risk factors in 1,019 subjects, aged 18-93 years. DNA damage was analyzed as strand breaks by the comet assay and levels of formamidopyrimidine (FPG-) and human 8-oxoguanine DNA glycosylase 1 (hOGG1)-sensitive sites There was an association between age and levels of FPG-sensitive sites for women, but not for men. The same tendency was observed for the level of hOGG1-sensitive sites, whereas there was no association with the level of strand breaks. The effect of age on oxidatively damaged DNA in women disappeared in multivariate models, which showed robust positive associations between DNA damage and plasma levels of triglycerides, cholesterol and glycosylated hemoglobin (HbA_1c_). In the group of men, there were significant positive associations between alcohol intake, HbA_1c_ and FPG-sensitive sites in multivariate analysis. The levels of metabolic risk factors were positively associated with age, yet only few subjects fulfilled all metabolic syndrome criteria. In summary, positive associations between age and levels of oxidatively damaged DNA appeared mediated by age-related increases in metabolic risk factors.

## INTRODUCTION

Aging is widely acknowledged to be associated with oxidative stress, typically referred to as the free radical theory of aging [1]. In addition, oxidative stress is implicated in age-related diseases such as cancer and cardiovascular diseases [2]. Oxidative stress-generated damage to DNA may be due to a declining activity of the antioxidant defense and repair systems or increased level of pro-oxidant factors, or both [3]. The most commonly investigated oxidative stress-generated DNA nucleobase lesion is 8-oxo-7,8-dihydroguanine (8-oxoGua) that is also one the most common pre-mutagenic lesions in the genome. This lesion can be measured by mass spectrometry after chromatographic isolation, although it is more common to measure the 2′-deoxyguanosine analogue 8-oxo-7,8-dihydro-2′-deoxyguanosine (8-oxodG) by either mass spectrometry or electrochemical detection. Alternatively, enzymic detection of oxidized purines, including 8-oxoGua by formamidopyrimidine DNA glycosylase (FPG) has been widely used in biomonitoring studies [4]. It has been shown that there is a positive association between age and increased level of FPG-sensitive sites in leukocytes from humans [5;6], whereas no associations between the level of FPG-sensitive sites and age, sex or smoking status have been reported in cross-sectional studies [7;8].

The DNA in human cells is oxidatively damaged by various endogenous biochemical processes, but exposure to hazardous environmental compounds and lifestyle factors are also important determinants for levels of oxidatively damaged DNA [4]. An excessive energy intake and sedentary lifestyle are associated with obesity and metabolic syndrome, which has high prevalence in both Europe and USA [9-11]. Metabolic syndrome is defined by the presence of a number of risk factors for cardiovascular diseases, including glucose intolerance, hypertension, hyperlipidemia, insulin resistance and obesity [12]. These and similar metabolic risk factors, such as increased levels of glycosylated hemoglobin (HbA_1c_), accumulate with age. It has been shown that hyperlipidemia is associated with elevated levels of oxidatively damaged DNA in peripheral blood mononuclear cells (PBMCs) [13]. Obesity, the metabolic syndrome and type 2 diabetes mellitus are associated with elevated levels of oxidative stress and low-grade inflammation [14]. Indeed, a positive association between the body mass index (BMI, i.e. the body weight in kilograms divided by the square of the height in meters) and elevated levels of FPG-sensitive sites in lymphocyte has been reported in a study of young and healthy subjects [15]. It has been shown that patients with type 2 diabetes mellitus or vascular disease had higher levels of 8-oxodG in lymphocytes than healthy subjects [16;17]. Another study showed that patients with essential hypertension had higher levels of 8-oxodG in leukocytes than subjects with normal blood pressure at the same age and body weight, although it should be noted that the statistical analysis did not control for metabolic or lifestyle factors [18]. Potential lifestyle factors of importance include a number of self-inflicted exposures such as alcohol intake, smoking and dietary components that may explain inter-individual variation in the basal level of DNA damage [19-22].

The objective of this study was to investigate the association between aging, metabolic risk factors and levels of oxidative damage to DNA in PBMCs from healthy subjects in the general population. In this respect, “healthy” implies that the subjects did not have specific diseases, although it is nowadays difficult to find subjects in the elderly population in countries with a proficient healthcare system, who are not “patients” in one way or another. We used PBMCs because they circulate through all organs of the body and they can be obtained from healthy humans. In addition, dysfunction of PBMCs is observed in age-related immune senescence, and aging-related telomere shortening in circling leukocytes have been observed in human cross-sectional and longitudinal studies [23;24]. The present study had a sufficient number of subjects to discriminate between aging *per se* and other contributing factors. As a rule of thumb, there should be approximately 100 observations for each independent variable in a multi-factorial analysis. We sampled blood from 1,019 subjects, allowing 10 independent variables in the statistical analysis. Our study was part of a cross-sectional study that had focus on lifestyle factors including diet, smoking, alcohol and physical activity [25]. The information about these variables was obtained by self-administered questionnaires, standard blood tests and physical performance tests. We hypothesized that there would be an association between age and levels of DNA damage in PBMCs which could be explained by metabolic risk factors.

## RESULTS

We analyzed the level of DNA damage in PBMCs from 1,019 subjects. There was missing information from the questionnaire data from 26 subjects. The effective study size was therefore 993 subjects, although there was also missing information for some of dependent variables in this dataset. The results on sex, obesity (BMI or waist-hip ratio (WHR, i.e. the ratio of body circumferences at the waist and the hip)), blood pressure, plasma lipids (cholesterol and triglycerides), HbA_1c_, C-reactive protein (CRP) smoking, alcohol intake and cardiorespiratory fitness in age-stratified groups, are shown in Table 1. In univariate linear regression analysis (Table 2), there were age-dependent positive associations in both sexes between age and BMI (P<0.001), WHR (P<0.001), cholesterol (P<0.001), triglycerides (P<0.001), HbA_1c_ (P<0.001), systolic blood pressure (P<0.001), diastolic blood pressure (P<0.001). There was an inverse association between age and cardiorespiratory fitness (P<0.001) and the CRP level was not associated with age (P>0.05).

**Table 1:** Metabolic, physiological and lifestyle variables of the study participants in age-stratified groups

Variable	Total	18-29 yr	30-49 yr	50-69 yr	70-93 yr
Sex (Men/women)	387/605	45/95	148/223	148/215	46/72
BMI (kg/m^2^)	24.2±0.11 (993)	22.2±0.21 (140)	23.8±0.16 (372)	24.9±0.20 (363)	25.6±0.33 (118)
WHR	0.87±0.01 (993)	0.82±0.01 (140)	0.85±0.01 (372)	0.89±0.01 (363)	0.91±0.01 (118)
Diastolic blood pressure (mmHg)	79±0.3 (993)	74±0.7 (140)	78±0.5 (372)	83±0.6 (363)	79±1.0 (118)
Systolic blood pressure (mmHg)	124±0.5 (993)	117±1.0 (140)	120±0.7 (372)	128±1.0 (363)	133±1.6 (118)
Mean arterial blood pressure (mmHg)	94±0.4 (993)	88±0.7 (140)	92±0.5 (372)	98±0.6 (363)	97±1.1 (118)
Cholesterol (mmol/L)	5.24±0.03 (993)	4.60±0.07 (140)	4.94±0.05 (372)	5.69±0.05 (363)	5.54±0.09 (118)
Triglycerides (mmol/L)	1.54±0.03 (993)	1.36±0.07 (140)	1.44±0.04 (372)	1.68±0.04 (363)	1.59±0.07 (118)
CRP (mg/L)	2.17±0.18 (993)	2.56±0.40 (139)	1.70±0.29 (372)	2.11±0.30 (363)	3.34±0.47 (118)
HbA_1c_ (%)	5.38±0.01 (991)	2.13±0.02 (139)	5.28±0.02 (371)	5.51±0.02 (363)	6.71±0.05 (118)
Smoking (Nonsmoker/smoker)	557/436	105/35	231/141	157/206	64/54
Alcohol intake (drinks per week)	10 ± 0.3 (971)	10±0.7 (135)	7 ± 0.3 (366)	12 ± 0.6 (357)	11±1.0 (113)
Cardiorespiratory fitness (mL/min/kg)	36±0.3 (722)	41±0.6 (133)	39±0.4 (338)	31±0.5 (224)	25±1.0 (27)

BMI: body mass index, WHR: waist-hip ratio, CRP: C-reactive protein. The values are mean ± SEM (number of subjects).

**Table 2:** Associations between age, metabolic and lifestyle factors and physiological variables

	Age	BMI	WHR	MAP	CHO	TRI	CRP	HbA_1c_	ALC	CRF
Age		0.25*	0.37*	0.36*	0.48*	0.20*	0.04	0.45*	0.09^§^	−0.56*
BMI	0.33*		0.45*	0.28*	0.19*	0.37*	0.12^#^	0.16*	−0.06	−0.51*
WHR	0.50*	0.61*		0.31*	0.29*	0.42*	0.13^#^	0.19*	0.06	−0.37*
MAP	0.24*	0.29*	0.28*		0.28*	0.26*	0.07	0.15*	0.03	−0.24*
CHO	0.30*	0.15^#^	0.16^#^	0.26*		0.29*	−0.03	0.31*	0.13^#^	−0.33*
TRI	0.04	0.25*	0.21*	0.13^§^	0.31*		0.11^#^	0.16*	−0.03	−0.29*
CRP	0.10	0.12^§^	0.11^§^	0.03	−0.02	−0.03		−0.01	0.03	−0.13^#^
HbA_1c_	0.36*	0.28*	0.29*	0.06	0.04	0.08	0.03		−0.02	−0.23*
ALC	0.22*	0.14^#^	0.22*	0.18*	0.07	0.02	−0.02	0.03		−0.03
CRF	−0.67*	−0.56*	−0.54*	−0.26*	−0.39*	−0.23*	−0.14^§^	−0.24*	−0.08	

The values are r-values of univariate linear regression analysis.

* P<0.001

# P<0.01

§ P<0.05

Values to the left and right of the shaded ladder are r-values in the strata of men and women, respectively. ALC: alcohol intake, BMI: body mass index, CHO: cholesterol, CRF: cardiorespiratory fitness, MAP: mean arterial blood pressure; TRI: triglycerides, WHR: waist-hip ratio.

**Figure 1:**
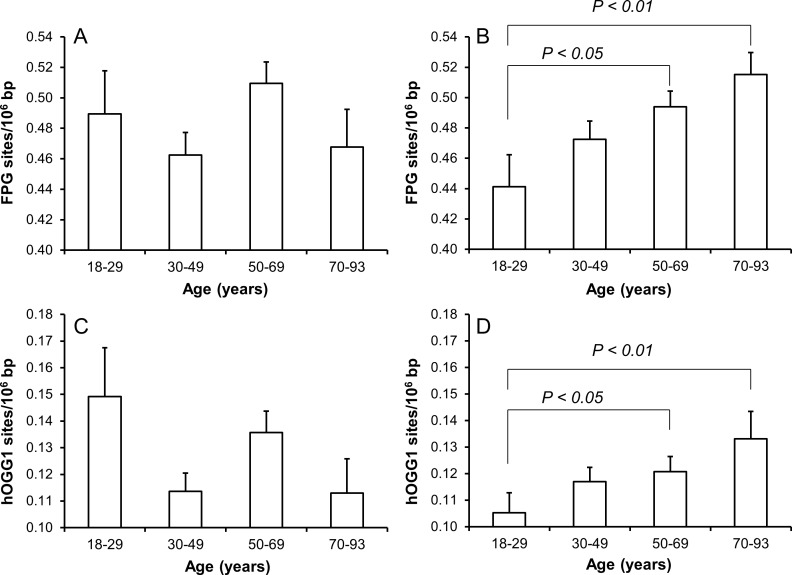
Levels of oxidatively damaged DNA in PBMCs from subjects in different age groups of men (A and C) and women (B and D) The number of subjects in the age groups are 45/95 (18-29 yr), 148/223 (30-49 yr), 148/215 (50-69 yr) and 46/72 (70-93 yr) for men/women. The bars and whiskers are means and SEM.

There were positive associations between age and levels of both FPG- and human 8-oxoguanine DNA glycosylase 1 (hOGG1)-sensitive sites for women (P<0.001), but not for men (P>0.05), in univariate linear regression analysis. Figure 1 shows the level of oxidatively damaged DNA in age-stratified groups. Both the FPG- and hOGG1-sensitive sites were elevated in the groups of women at the age of 50-69 yr (P<0.05, ANOVA) and 70-93 yr (P<0.01, ANOVA) as compared to the women in the youngest age group. There was no association between age and levels of DNA strand breaks (Supplementary Figure 1).

**Figure 2:**
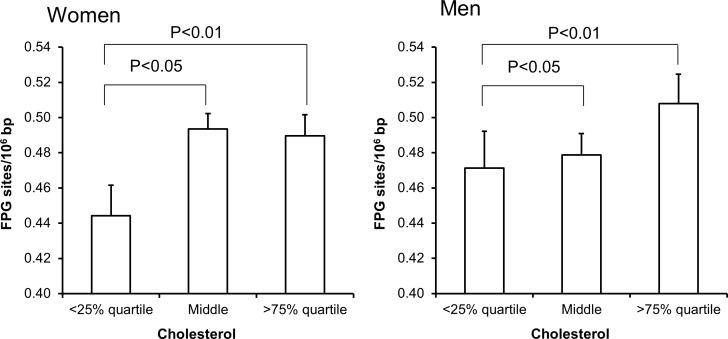
Levels of FPG-sensitive sites in PBMCs from subjects stratified in plasma cholesterol concentrations being less than the 25% quartile, middle or more the 75% quartile for the sex These groups were <4.58 (n = 150), 4.58-5.95 (n = 303) and >5.95 (n = 152) for the women. For men, these groups were <4.39 (n = 94), 4.39-5.77 (n = 196) and >5.77 (n = 97). The bars and whiskers are means and SEM. The P-values correspond to ANOVA tests with group as categorical variable.

**Figure 3:**
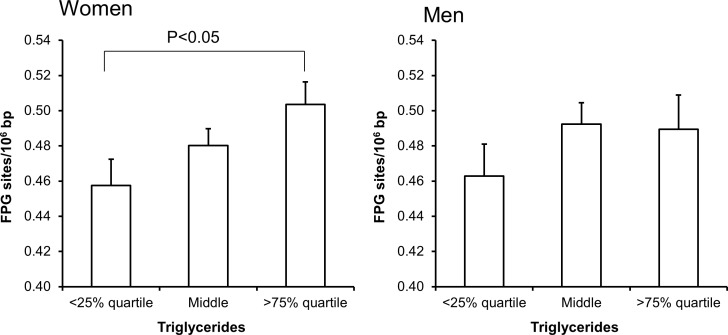
Levels of FPG-sensitive sites in PBMCs from subjects stratified into groups of plasma triglycerides concentrations being less than the 25% quartile, middle or more the 75% quartile for the sex These groups were <0.95 (n = 151), 0.95 – 1.67 (n = 303) and >1.67 (n = 151) for the women. For men, these groups were <1.11 (n = 97), 1.11 – 2.07 (n = 194) and >2.07 (n = 96). The bars and whiskers are means and SEM. The P-values correspond to ANOVA tests with group as categorical variable.

**Figure 4:**
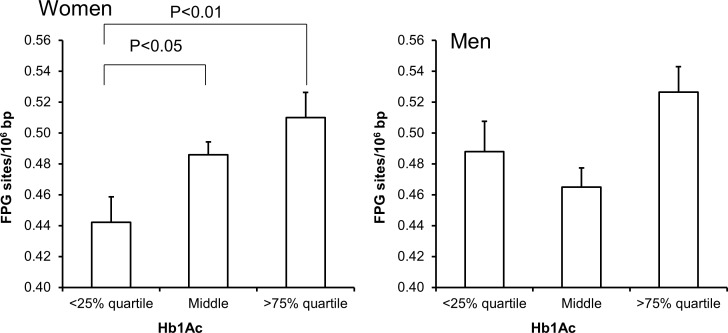
Levels of FPG-sensitive sites in PBMCs from subjects stratified into plasma levels of HbA being less than the 25% quartile, middle or more the 75% quartile for the sex These groups were <5.2 (n = 137), 5.2 – 5.6 (n = 367) and >5.6 (n = 99) for the women. For men, these groups were <5.2 (n = 86), 5.2 – 5.6 (n = 212) and >5.5 (n = 89). The bars and whiskers are means and SEM. The P-values correspond to ANOVA tests with group as categorical variable.

**Figure 5:**
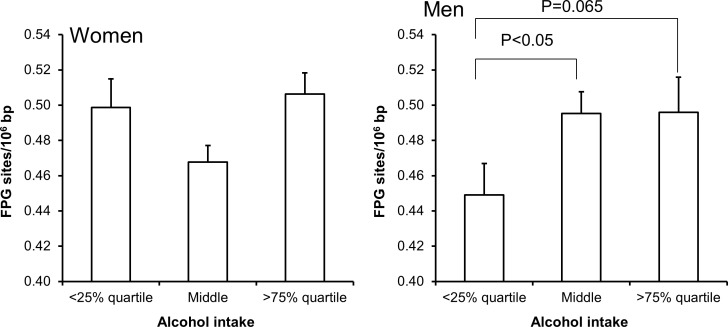
Levels of FPG-sensitive sites in PBMCs from subjects stratified into groups of alcohol intake being less than the 25% quartile, middle or more the 75% quartile for the sex These groups were <2 (n = 111), 2 – 11 (n = 345) and >11 (n = 133) for the women. For men, these groups were <5 (n = 90), 5 - 19 (n = 199) and >19 (n = 92). The bars and whiskers are means and SEM. The P-values correspond to ANOVA tests with group as categorical variable.

**Figure 6:**
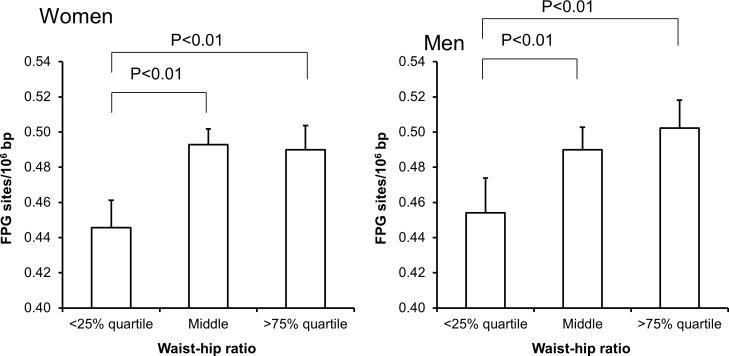
Levels of FPG-sensitive sites in PBMCs from subjects stratified into WHR level being less than the 25% quartile, middle or more the 75% quartile for the sex These groups were <0.78 (n = 151), 0.78 – 0.87 (n = 303) and >0.87 (n = 151) for the women. For men, these groups were <0.87 (n = 95), 0.87 – 0.97 (n = 196) and >0.97 (n = 96). The bars and whiskers are means and SEM. The P-values correspond to ANOVA tests with group as categorical variable.

Univariate regression analysis showed that the level of FPG-sensitive sites was associated with levels of cholesterol, triglycerides, HbA_1c_ and alcohol intake in either one or both sexes (Table 3). The multivariate regression analysis showed associations between FPG-sensitive sites and plasma levels of cholesterol (P<0.01) and triglycerides (P<0.05) among women. Figure 2 and 3 show the level of FPG-sensitive in groups of subjects who have been stratified for plasma level of cholesterol and triglycerides, respectively. The linear regression analysis indicated associations between FPG-sensitive sites and HbA_1c_ in both men (P<0.05) and women (P<0.001), although it is clear that the association was strongest in the women as also indicated on the ANOVA test (Figure 4). The association between FPG-sensitive sites and alcohol intake was strongest among men (P<0.05). Figure 5 shows the level of FPG-sensitive sites in subjects who have been stratified into groups according to the level of alcohol intake. There was also a modest positive association in the whole group of subjects between the level of WHR and FPG-sensitive (P<0.05), although this was not robust in multivariate regression analysis. Figure 6 shows the level of FPG-sensitive sites in groups of subjects stratified according to the level of WHR; the difference in WHR was statistically significant in the overall ANOVA (P<0.001), whereas there was no association between BMI and levels of FPG-sensitive sites (Supplementary Figure 2).

**Table 3:** Results of regression analysis for age and metabolic and lifestyle factors as predictors of the level of FPG-sensitive sites

Variable	All	Men	Women
Age	0.99 (0.33)^#^	0.37 (0.56)	1.36 (0.41)*
Sex	2.57 (11.1)	NA	NA
BMI	2.04 (1.56)	−1.09 (2.78)	3.63 (1.92)
WHR	127 (65)^§^	180 (120)	161 (105)
MAP	0. 27 (0.33)	0. 51 (0.59)	0. 14 (0.40)
Cholesterol	**16.0 (5.5)**^#^	12.4 (8.9)	18.8 (7.0)^#^
Triglycerides	13.6 (6.6)^§^	5.89 (9.24)	23.2 (9.7)^§^
HbA1c	52.6 (13.4)*	**42.5 (19.2)**^§^	63.3 (19.0)*
CRP	0.87 (0.99)	0.28 (1.53)	1.35 (1.32)
Smoking	−3.72 (10.9)	−6.47 (18.0)	−2.27 (13.9)
Alcohol	**1.30 (0.60)**§	**1.88 (0.86)**^§^	**0.72 (0.93)**

The data are reported as slope and standard error (per 10^3^).

§ P<0.05

# P<0.01

* P<0.001 (univariate linear regression analysis)

Bold numbers indicate variables with statistically significantly effect in stepwise forward and backward multi-variable regression analysis. BMI: body mass index, WHR: waist-hip ratio, MAP: mean arterial blood pressure, CRP: C-reactive protein, NA: not applicable.

**Figure 7:**
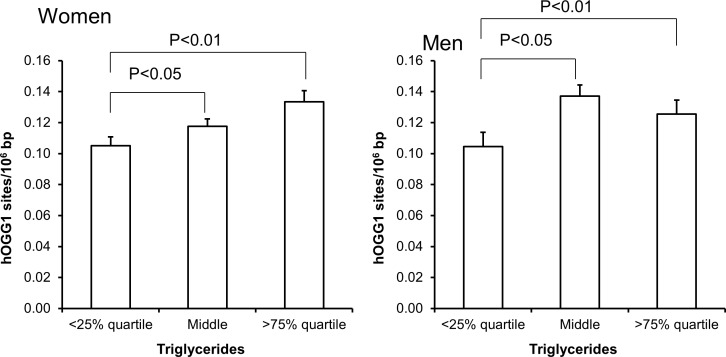
Levels of hOGG1-sensitive sites in PBMCs from subjects stratified into serum levels of triglycerides being less than the 25% quartile, middle or more the 75% quartile for the sex These groups were <0.95 (n = 151), 0.95 – 1.67 (n = 303) and >1.67 (n = 151) for the women. For men, these groups were <1.11 (n = 97), 1.11 – 2.07 (n = 194) and >2.07 (n = 96). The bars and whiskers are means and SEM. The P-values correspond to ANOVA tests with group as categorical variable.

The level of hOGG1-sensitive sites was associated with age in women (Figure 1), whereas there only was association between the level of hOGG1-sensitive sites and plasma concentration of triglycerides in univariate regression analysis (Table 4). This association remained statistically significant in multivariate regression analysis in both sexes (P<0.05, Table 4). However, the association was mainly driven by the association among the women, whereas the ANOVA test on categorized triglyceride levels also indicated positive associations between plasma triglyceride levels and hOGG1-sensitive sites among men (Figure 7). There were no associations between levels of hOGG1-sensitive sites and BMI (Supplementary Figure 3) or WHR (Supplementary Figure 4).

**Table 4:** Results of regression analysis for age and metabolic and lifestyle factors as predictors of the level of hOGG1-sensitive sites

Variable	All	Men	Women
Age	0.27 (0.17)	−0.03 (0.30)	0.44 (0.20)^§^
Sex	7.55 (5.66)	NA	NA
BMI	0.87 (0.79)	0.21 (1.50)	0.93 (0.93)
WHR	39.6 (32,7)	−7.29 (64.4)	44.6 (50.5)
MAP	0.31 (0.17)	0.12 (0.31)	0.35 (0.19)
Cholesterol	0.80 (2.8)	−1.71 (4.80)	3.00 (3.39)
Triglycerides	**7.52 (3.32)**^§^	3.46 (4.97)	**11.3 (4.69)**^§^
HbA1c	9.12 (6.84)	1.74 (10.3)	16.9 (9.23)
CRP	0.23 (0.50)	1.05 (0.82)	−0.46 (0.63)
Smoking	−2.85 (5.59)	−17.1 (9.61)	5.63 (6.75)
Alcohol	0.14 (0.31)	−0.03 (0.47)	0.11 (0.45)

The data are reported as slope and standard error (per 10^3^).

§ P<0.05 (univariate linear regression analysis).

Bold numbers indicate variables with statistically significantly effect in stepwise forward and backward multi-variable regression analysis. BMI: body mass index, WHR: waist-hip ratio, MAP: mean arterial blood pressure, CRP: C-reactive protein, NA: not applicable.

There was no difference between subjects, who fulfilled all defined criteria for metabolic syndrome, and controls in terms of DNA strand breaks (0.18 ± 0.01 versus 0.18 ± 0.02 lesions/10^6^ bp, P=0.34), FPG-sensitive sites (0.52 ± 0.02 versus 0.50 ± 0.02 lesions/10^6^ bp, P=0.97), and hOGG1-sensitive sites (0.12 ± 0.01 versus 0.13 ± 0.01 lesions/10^6^ bp, P=0.80), respectively.

## DISCUSSION

**Figure 8:**
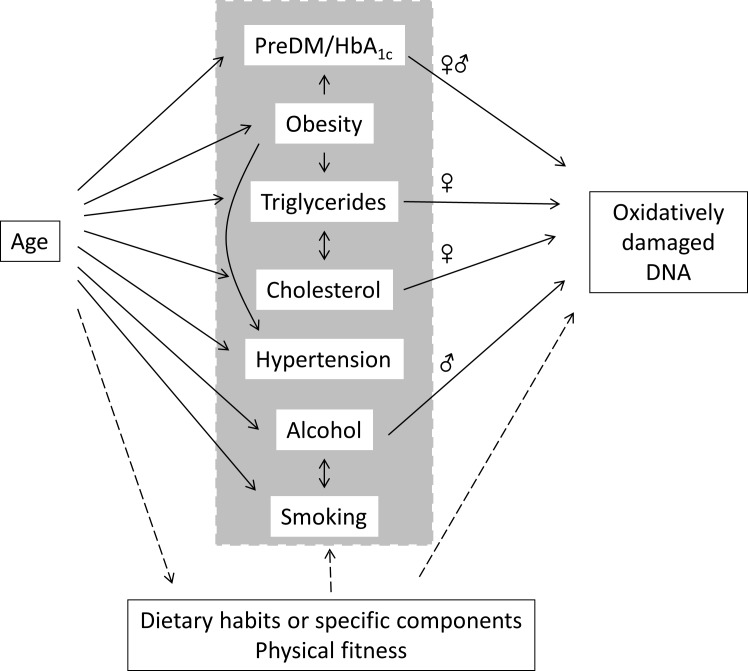
Associations between age, mediators and oxidatively damaged DNA in PBMCs in the present study Arrows represent pathways with the strongest statistically significant effect in univariate models. The sex symbols (♀ or ♂) indicate associations between pre-diabetes mellitus (preDM/HbA_1c_), triglycerides, cholesterol or alcohol intake is either both or only one sex strata.

In this study we showed an association between age and levels of oxidatively damaged DNA particularly in women, whereas the same trend was not significant among men. Figure 8 depicts a schematic overview of the associations between age and oxidatively damage DNA in PBMCs, highlighting the effects of possible mediators. There were associations between age and blood pressure, obesity, plasma lipids and HbA_1c_ and alcohol intake, whereas the cardiorespiratory fitness was inversely associated with age. The association between age and oxidatively damaged DNA disappeared in multivariate regression analysis models, which showed robust associations between levels of oxidatively damaged DNA and measures of plasma lipids, HbA_1c_ and alcohol intake. These results indicate that the positive association between age and level of oxidatively damaged DNA was mediated by metabolic and lifestyle factors rather than the aging process *per se*. The multivariate analysis also indicated that measures of blood pressure and obesity were not associated with the level of oxidatively damaged DNA.

The results demonstrate sex differences in age-related levels of DNA damage and effect of potential mediators. This is most likely based on multiple factors, some of which are related to specific exposures. For instance, the strongest effect of alcohol intake was observed in the strata of men, which had both higher mean intake and standard deviation (12.6 ± 10.7 drinks per week) as compared to women (7.4 ± 7.5 drinks per week). The effect of metabolic risk factors may also display sex-dependent trends because there are biological differences between men and women. However, it should also be acknowledged that most of the metabolic risk factors are proxy-measures of biological features. For instance, both BMI and WHR are descriptors of obesity, but the r-values between BMI and WHR were only 0.61 and 0.45 in men and women in the present dataset, respectively. The relatively low correlation coefficient between BMI and WHR indicates that there are several ways of being obese and this may be sex-dependent. The stronger effect of metabolic risk factors in the strata of women might be due to the larger number of women in the dataset, although this should not affect the size of regression coefficients. Finally, sex-specific interactions between metabolic risk factors might explain why some trends are stronger displayed only in one sex. For instance, plasma levels of triglycerides and cholesterol correlate significantly in both men and women, whereas there is only a significant correlation between these plasma lipids and HbA_1c_ in men. We have not sought to unravel interactions between metabolic risk factors and levels of DNA damage because the dataset most likely will be statistically under-powered in such analysis.

It has been shown that circulating PBMCs from obese subjects displayed a pro-inflammatory phenotype [26]. A pro-inflammatory state in obesity can be associated with oxidative stress by for instance increased activity NADPH oxidase [27]. We observed a positive association between CRP levels and obesity measures (BMI or WHR) in univariate linear regression model. However, there were relatively few subjects in the present study who were obese (BMI > 30 kg/m^2^) and there were only weak associations between measures of obesity and oxidatively damaged DNA in PBMCs. A previous study showed a positive association between BMI and FPG-sensitive sites in lymphocytes of non-obese women aged 19-31 years, whereas there were no association in men [15]. However, a cross-sectional study of mainly non-obese subjects in Florence, Italy, showed no association between the BMI and FPG-sensitive sites [20].

Our results point to an effect of the plasma levels of triglycerides, rather than obesity, on the level of oxidatively damaged DNA in PBMCs. The positive association between the level of triglycerides and oxidatively damaged DNA in PBMCs was robust after adjustment for variables related to obesity (BMI and WHR). It indicates that the association could be related directly to an effect of the lipids, rather than the level of triglycerides being a proxy-measure of a non-lipid variable with DNA damaging potential. This is supported by a study of patients with hyperlipidemia who had a positive correlation between levels of 8-oxodG in PBMCs and plasma levels of triglycerides and insulin resistance, whereas there were no correlation with BMI and abdominal circumference [13]. The measurement of triglycerides in plasma mainly represents lipids stored in lipoproteins, which is considered to be less hazardous than non-esterified free fatty acids [28;29]. Nevertheless, it has been shown that there is a correlation between triglycerides and non-esterified fatty acids in women, whereas no such association was observed in men [30]. This could explain the stronger association between plasma lipids and oxidatively damaged DNA in our study. Another study showed a positive correlation between DNA strand breaks, although not FPG-sensitive sites, in PBMCs and concentrations palmitic acid, decosahexaenoic acid, linoleic acid and oleic acid in erythrocytes [31]. The non-esterified free fatty acids in blood originate from lipolysis in adipocytes and elevated levels of these lipids have been associated with hepatic oxidative stress and insulin resistance [32]. This is supported by experimental studies showing that infusion of triglycerides in the circulation of healthy humans was associated with oxidative stress and a pro-inflammatory state in PBMCs [33].

The linkage between high blood glucose levels and oxidative damage to DNA in lymphocytes is well established for diabetics [16]. In addition, it has been described that there is an association between the glycemic status and oxidatively damaged DNA in PBMCs in well-controlled diabetes patients [34]. We used the plasma HbA_1c_ as a marker of the average blood glucose concentration during the last three months. The Danish threshold for type 2 diabetes is 6.5% HbA_1c_ (corresponding to 48 mmol/mol or a mean glucose level of 7.7 mmol/L), whereas a level of 6.0-6.5% is considered to be an indication of pre-diabetic status. By this standard there were 13 subjects (1.3%) having pre-diabetic status or type 2 diabetes mellitus in our study. Nevertheless, our study shows a robust association between the level of HbA_1c_ and FPG-sensitive sites below the threshold of pre-diabetes, suggesting that this can be part of the mediation of age-related accumulation of DNA damage.

The strongest associations (i.e. highest slopes in the regression analysis) between metabolic risk factors and levels of oxidatively damaged DNA were observed in the strata of women for obesity, HbA_1c_, triglycerides and cholesterol. Interestingly, a previous study showed no difference in levels of hOGG1-sensitive sites in leukocytes of age-matched (average 47 years) subjects with metabolic syndrome and controls [35]. It has been shown that obesity and diabetes are associated with risk of cancer is several organs and the risk is sex-dependent [36;37]. Likewise, blood glucose and triglycerides levels have been associated with increased risk of cancers that differ between the sexes [38;39], whereas elevated serum levels of cholesterol has been shown to be inversely associated with cancer risk [40]. It should be noted that these epidemiological studies have not adjusted for other important types of metabolic risk factors and residual confounding is possible. However, it worthwhile noting that the different strength of association and sex-related differences between metabolic risk factors and levels of oxidatively damaged DNA, is also observed in epidemiology on disease endpoints.

The subjects in the present study were relatively fit physically and healthy. It has been shown that men with low aerobic fitness had high age-related accumulation in FPG-sensitive sites in lymphocytes (r = 0.74) as compared with men with high aerobic fitness (r = 0.32), assessed by VO_2_max measurements [41]. However, a positive correlation between VO_2_max and levels of FPG-sensitive sites in PBMCs has also been observed in a cross-sectional study on subjects aged 20 to 84 years [42]. Nevertheless we have found no association between physical performance (determined as maximal jump force, handgrip strength and number of rises from a chair in 30 sec) and FPG-sensitive sites in PBMCs from the middle-aged, all born in 1953 in the Copenhagen Metropolitan area [43]. The level of physical fitness in the study populations may explain the apparent discrepancy between studies showing no effect of age and age-related accumulation in oxidatively damaged DNA, although this parameter is not usually assessed in cross-sectional studies.

Diet can be regarded as a complex mixture of components having beneficial (e.g. antioxidants) or hazardous mechanisms (pro-oxidant compounds). We have not assessed the effect of specific dietary items in this study. It should be acknowledged that dietary habits (or specific components) may be uncontrolled confounders as well as mediators. It has been shown that the level of 8-oxodG in leukocytes was positively associated with age (20-90 years) and it correlated positively with some plasma antioxidants (vitamin E and uric acid) and negatively with other (ascorbic acid) antioxidants [44]. Observations from a cross-sectional study with 340 subjects in Oslo, Norway, showed an inverse association between levels of FPG-sensitive sites in PMBCs and habitual fruit intake, whereas there were no significant associations with a number of biomarkers of antioxidant status in plasma [45]. A cross-sectional study on 201 inhabitants in Bremen, Germany, found no association between the level of FPG-sensitive sites and age, sex or smoking status [7]. Nevertheless, several intervention studies have shown that supplementation with antioxidant-rich food items was associated with reduced levels of oxidatively damaged nucleobase products in leukocytes and urine [46;47]. However, dietary interventions with substitution to food rich in fruits and vegetables had little effect on the level of oxidatively damaged DNA in PBMCs [48;49]. Even though associations are at best weak between oxidatively induced DNA damage and a diet rich in fruits and vegetable, which would also be expected to reduce obesity, plasma lipids and Hb1Ac, we cannot exclude confounding from that aspect of diet.

The positive association between age and levels of oxidatively damaged DNA is in keeping with the notion that aging is associated with oxidative stress because of reduced activity of the antioxidant system or increased production of reactive oxygen species [1;2] and this could partly by mediated by metabolic factors as suggested by our study. There is compelling evidence from animal experimental models that the levels of oxidatively damaged DNA in tissues are associated with the age [50], which could be a pro-oxidant effect as well as a reduced activity of the DNA repair system [51]. The studies on aging in small rodents are relatively straightforward because of the short lifespan, whereas the long lifespan of humans typically allows only cross-sectional types of studies as our study. In such a design, one compares subjects with different age and possibly also different lifestyles. In addition, selection bias is difficult to avoid because of voluntary participation. This is also an issue in the present study, considering limited participation (11.6% of all invited persons in the area), suggesting a selection bias toward more health conscious participants. However, the strength of our study is the large number of subjects, which makes it possible to assess some of the age-related lifestyle and metabolic factors as mediators of DNA damage.

In summary, there was an association between age and levels of FPG- and hOGG1-sensitive sites in particular among women, whereas the association was not significant for men. The accumulation of DNA damage with age could be mediated by metabolic factors including plasma lipids and HbA_1c_ as well as intake of alcohol which was positively associated with age and showed significant robust positive associations with the levels of oxidatively-induced DNA damage.

## METHODS

### Sampling

We collected blood samples and lifestyle related data from 1,019 subjects in Copenhagen during a national survey of the health status in thirteen different municipalities around Denmark [25]. Our collection of samples took place in September 2008 among 1,739 participants in the municipality of Frederiksberg which had a participation rate of 11.6% of the invited adult population. The sampling for the present study ceased after having collected blood from the first thousand subjects. Approximately 100 subjects were invited to participate per day and were subjected to a full health examination, including anthropometric and other health related measurements. There were no inclusion or exclusion criteria in our sampling, which included randomly selected men and women aged 19-93 years. We obtained 7 mL venous blood in cell preservation tubes (Vacutainer, Becton Dickinson A/S, Brøndby, Denmark) from each subject, isolated and cryopreserved the PBMCs (for comet assay) in a solution with 50% fetal bovine serum, 40% RPMI medium and 10% dimethyl sulphoxide as described in a previous study [52].

From the questionnaires, we obtained information about age, sex, alcohol intake and smoking status. The physical examination included determination of BMI, WHR, blood pressure and cardiorespiratory fitness tested on an ergometer bike as an indirect maximal or sub-maximal exercise test [25]. Non-fasting venous blood plasma samples were analyzed for cholesterol, triglyceride, CRP and HbA_1c_ at the Department of Clinical Biochemistry, KB 3-01-1, Rigshospitalet, Copenhagen University Hospital.

### Oxidatively damaged DNA

The level of DNA damage in PBMCs was analyzed by the single cell gel electrophoresis (comet) assay as strand breaks, FPG- and hOGG1-sensitive sites. The FPG- and hOGG1-sensitive sites are oxidatively damaged DNA in terms of ring-opened formamidopyrimidine lesions and 8-oxodG, whereas the FPG enzyme can also detect non-oxidatively generated DNA lesions following exposure to high doses/concentrations of alkylating agents [53;54].

A 150 μL of the cryopreserved PBMC suspension was mixed with 1000 μL low melting point agarose (0.75 % in PBS, Sigma-Aldrich) at 37 ^o^C and 60 μL was applied to GelBonds (Cambrex, Medinova Scientific A/S, Hellerup, Denmark). The Gelbonds were transferred to lysis solution (2.5 M NaCl, 100 mM Na_2_EDTA, 10 mM Trizma base, pH 10.0) for at least one hour. The FPG enzyme (1 mg/mL, gift from Professor Andrew Collins, University of Oslo, Norway), hOGG1 (0.16 units/gel, Medinova Scintific A/S, Denmark (P/N M0241L)) or a solution consisting of 40 mM HEPES, 0.1 M KCl, 0.5 mM Na_2_EDTA, 200 μg/mL BSA, pH 8.0 was applied onto the gels and they were covered with cover slip and incubated for 45 min at 37 ^o^C in a humidified box. The Gelbonds were transferred to the electrophoresis chamber containing an alkaline solution (1 mM Na_2_EDTA, 300 mM NaOH, pH 13.0) and left for 40 min at 4 ^o^C. Electrophoresis time was 20 min (25 V, 300 mA, 0.83 V/cm) in the same solution as alkaline unwinding treatment. The samples subsequently were neutralized by three times 5 min washing in neutralization buffer (0.4 M Trizma base, pH 7.5), rinsed with milliQ^®^ water and dried in 96 % ethanol.

We had reference control samples in each experiment (corresponding to one electrophoresis) that included one aliquot of PBMCs that had been exposed to the photosensitizer Ro19-8022 and white light, which generates high levels of FPG-sensitive sites. The Ro19-8022 photosensitizer was a kind gift from F. Hoffmann-La Roche (Basel, Switzerland). The mean ± standard deviation of the controls were 0.36 ± 0.17, 0.59 ± 0.20 and 1.07 ± 0.17 lesions/10^6^ bp for levels of DNA strand breaks, hOGG1- and FPG-sensitive sites, respectively.

The dried samples was stained with YOYO-1 iodide (P/N 491/509, Molecular Probes, The Netherlands) in PBS and 100 images per slide was scored (200 images per subject) using an Olympus fluorescence microscope at 40x magnification by using a five-class scoring system (arbitrary score range: 0-400). These scores were transformed to lesions per 10^6^ base pairs (bp) by means of a calibration curve based on induction of strand breaks by ionizing radiation, which has a known yield. We used a conversion factor of 0.0298 Gy equivalents per score and calculations were based on the assumption that an average molecular weight of a DNA bp is 650 Dalton [55]. The number of FPG- or hOGG1-sensitive sites was obtained as the difference in scores of parallel slides incubated with FPG, hOGG1 or buffer, respectively.

### Statistics

We used univariate and multivariate regression models including the following independent variables: age, sex, smoking, alcohol intake, obesity (BMI or WHR), cardiorespiratory fitness, blood pressure, plasma levels of lipids (cholesterol or triglycerides), HbA_1c_ and CRP with strand breaks, FPG- and hOGG1-sensitive sites as dependent variables. The associations between the independent variables were assessed in univariate regression models. We used the mean arterial blood pressure (MAP) in the statistical models because the systolic and diastolic blood pressure was strongly correlated (r = 0.71, P<0.001, linear regression). This was defined as: MAP = [(2 x diastolic pressure) + systolic pressure]/3. The statistical models included either BMI or WHR as measure of obesity because these were correlated (r = 0.51, P<0.001, linear regression). We have included only BMI in the multivariate regression models. The level of cardiorespiratory fitness (i.e. the maximal oxygen consumption VO_2_max determined in an exercise test) has been assessed as a descriptive variable of the subject's health status, whereas it is not included in multivariate regression analysis because of possible selection bias in the elderly. The health examination included also measurements of hand grip strength with observations in 993 subjects; in comparison there were measurements of VO_2_max in 722 subjects. The group of subjects who had not done the exercise test had a stronger inverse association between age hand grip strength test as compared to the group of subjects who had done the exercise test (P<0.01 for interaction between age and performance of exercise test).

The multivariate linear regression analysis included the age, sex, MAP, BMI, cholesterol, triglycerides, CRP, HbA_1c_, alcohol intake and smoking status with both forward and backwards variable selection. Variables that were statistically significant in multivariate regression analysis were also analyzed in ANOVA tests on categorized data (age, cholesterol, triglycerides and alcohol intake). The groups were defined as low (less than the 25% quartile), middle, and high (more than the 75% quartile) of the determining variable. The effect of the metabolic syndrome was assessed by stratifying the subjects into two categories with or without this condition. The criteria used for identifying subjects with metabolic syndrome were a modified version of the WHO standards [56]. The cut-off values defined by the WHO on three main criteria; obesity, hypertension and triglycerides were applied as follows. Obesity; BMI >30 kg/m^2^ or WHR >0.90 for men and >0.85 for female. Hypertension; systolic pressure >140 mmHg, diastolic pressure >90 mmHg, triglycerides ≥1.7 mmol/L. Subjects were identified as having metabolic syndrome when they expressed elevated values in all three criteria. There were relatively few subjects with metabolic syndrome and it seemed to depend on both age and sex. We therefore assessed the effect of metabolic syndrome in subjects with a control group that was matched for age, sex (with a six years span) and day of analysis of DNA damage. Thus selection yielded 94 pairs of subjects and the effect of metabolic syndrome was analyzed by the Students t-test.

All statistical analysis were performed in SAS institute 9.1.9 or Statistical version 5.5 from StatSoft, Inc., Tulsa, OK, USA and statistical significance was defined as P< 0.05.

## SUPPLEMENTARY MATERIAL FIGURES



## Abbreviations

BMI: body mass index
CRP: C-reactive protein
MAP: mean arterial pressure
PBMC: peripheral blood mononuclear cell
8-oxoGua: 8-oxo-7,8-dihydroguanine
8-oxodG: 8-oxo-7,8-dihydro-2′-deoxyguanosine
FPG: formamidopyrimidine DNA glycosylase
OGG1: 8-oxoguanine DNA glycosylase 1
WHR: waist-hip ratio
