# Supplementary material for: Age and metabolic risk factors associated with oxidatively damaged DNA in human peripheral blood mononuclear cells
Source: Oncotarget. 2014 Dec 18;6(5):2641–53. doi: 10.18632/oncotarget.3202 (PMC4413607; doi:10.18632/oncotarget.3202)
Supplement: Supplementary file 1 [file oncotarget-06-2641-s001.pdf]

Age and metabolic risk factors associated with oxidatively damaged DNA in human peripheral blood mononuclear cells

Supplementary Material

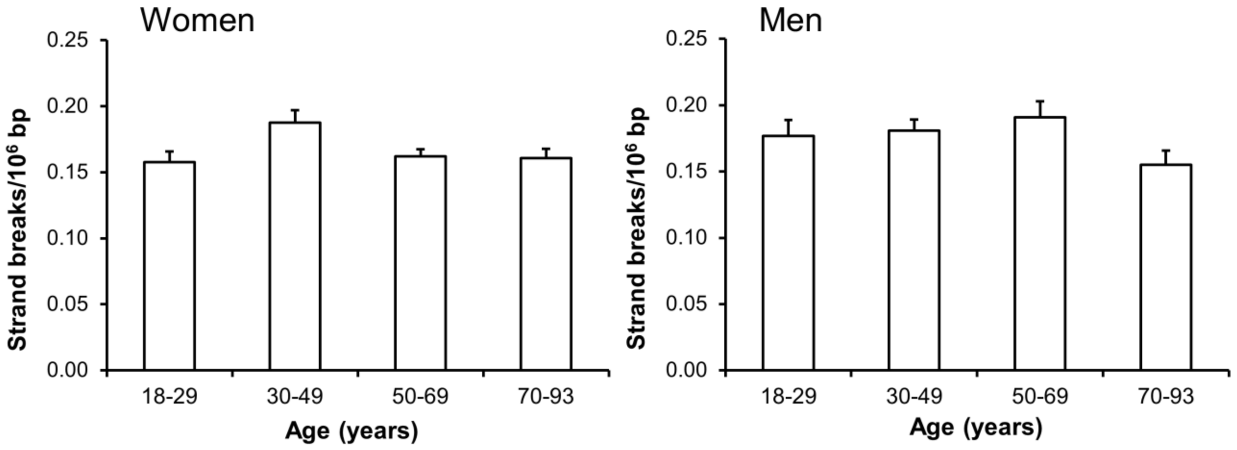

Supplementary figure 1: Levels of DNA strand breaks in PBMCs from subjects in different age groups of men and women. The number of subjects in the age groups are 45/95 (18-29 yr), 148/223 (30-49 yr), 148/215 (50-69 yr) and 46/72 (70-93 yr) for men/women. The bars and whiskers are means and SEM.

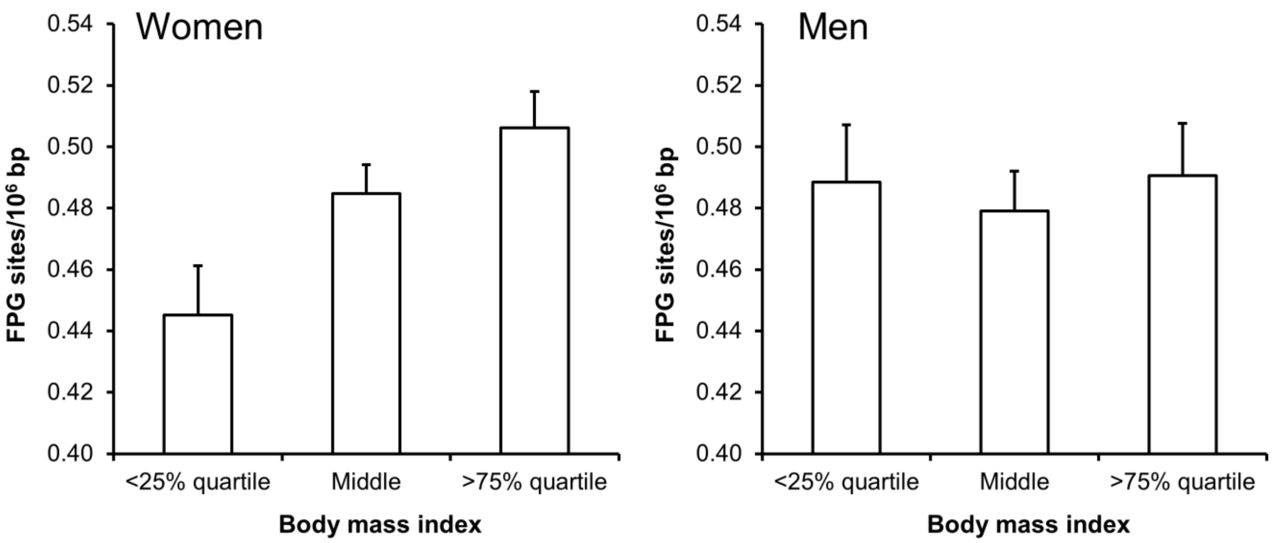

Supplementary figure 2: Levels of FPG-sensitive sites in PBMCs from subjects stratified into BMI level being less than the 25% quartile, middle or more the 75% quartile for the sex. These groups were <21.0 (n = 149), 21.0 – 25.4 (n = 305) and >0.25.4 (n = 151) for the women. For men, these groups were <22.7 (n = 96), 22.7 – 27.0 (n = 195) and >27.0 (n = 96). The bars and whiskers are means and SEM.

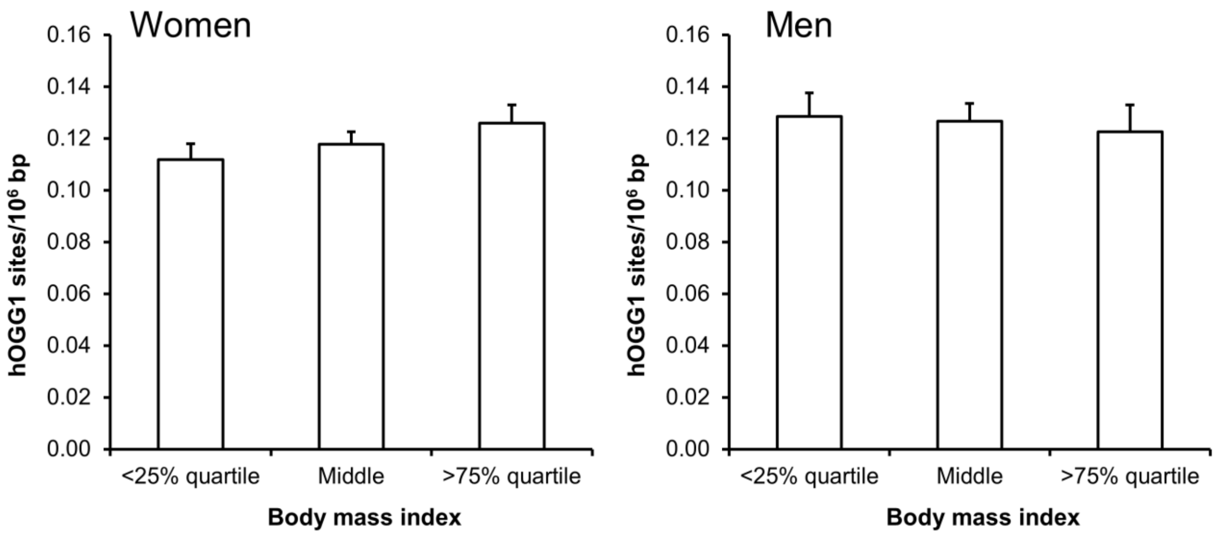

Supplementary figure 3: Levels of hOGG1-sensitive sites in PBMCs from subjects stratified into BMI level being less than the 25% quartile, middle or more the 75% quartile for the sex. These groups were <21.0 (n = 149), 21.0 – 25.4 (n = 305) and >0.25.4 (n = 151) for the women. For men, these groups were <22.7 (n = 96), 22.7 – 27.0 (n = 195) and >27.0 (n = 96). The bars and whiskers are means and SEM.

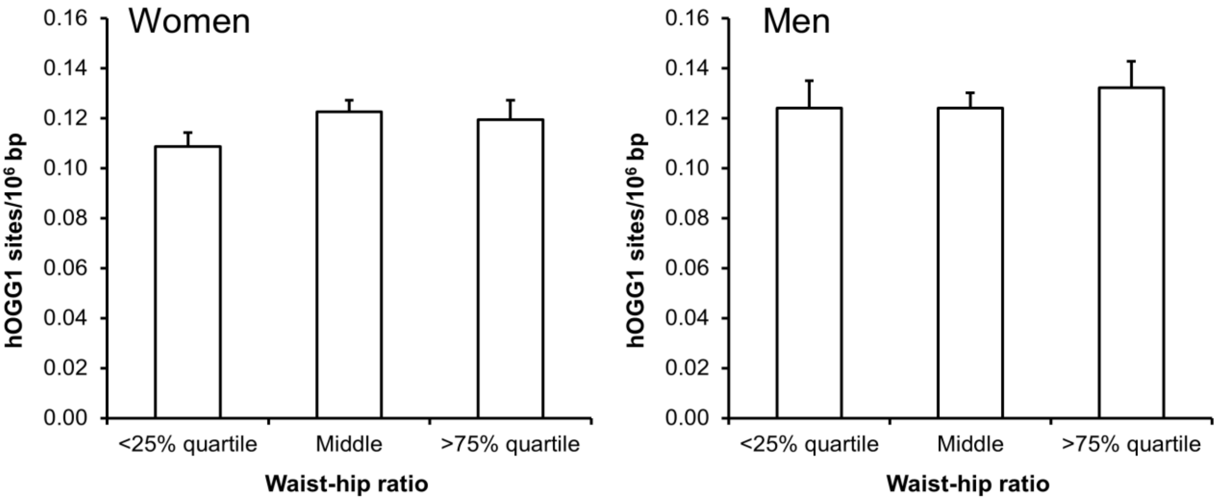

Supplementary figure 4: Levels of hOGG1-sensitive sites in PBMCs from subjects stratified into hip-waist level being less than the 25% quartile, middle or more the 75% quartile for the sex. These groups were <0.78 (n = 151), 0.78 – 0.87 (n = 303) and >0.87 (n = 151) for the women. For men, these groups were <0.87 (n = 95), 0.87 – 0.97 (n = 196) and >0.97 (n = 96). The bars and whiskers are means and SEM.
